# Efficient Hydrogen and Oxygen Evolution Catalysis Using 3D-Structured Nickel Phosphosulfide Nanosheets in Alkaline Media

**DOI:** 10.3390/molecules28010315

**Published:** 2022-12-30

**Authors:** Lei Lin, Qiang Fu, Junbei Hu, Ran Wang, Xianjie Wang

**Affiliations:** 1 Harbin Institute of Technology, School of Physics, Harbin 150001, China; 2 National Key Laboratory of Science and Technology on Advanced Composites in Special Environments, Harbin Institute of Technology, Harbin 150001, China

**Keywords:** water splitting, Ni-based nanomaterials, plasma surface treatment, vacancy defect, hydrogen evolution reaction, oxygen evolution reaction

## Abstract

Water electrolysis offers a zero-carbon route to generate renewable energy conversion systems. Herein, a self-supported nickel phosphosulfide nanosheet (NS) electrocatalyst was fabricated at a low temperature on carbon cloth, which was then subjected to Ar etching to enhance its catalytic activity. Etching resulted in better hydrogen evolution reaction (HER) and oxygen evolution reaction (OER) performance than other samples, with overpotentials of 103.1 mV (at 10 mA cm^−2^) and 278.9 mV (at 50 mA cm^−2^), respectively. The characterization results confirmed that Ar etching created a thin amorphous layer around the NiPS_3_ NSs, which increased the number of active sites and modulated their electronic structures. These 3D-structured NiPS_3_ NSs and their subsequent Ar etching process show promise for applications in overall water splitting in alkaline media.

## 1. Introduction

Clean energy has attracted extensive research attention in the past few decades due to the continuous growth of environmental pollution and energy demands. Hydrogen is a promising alternative to fossil fuels. The electrolytic reduction of water provides a zero-carbon route to clean and sustainable hydrogen resources. Electrochemical water splitting involves the cathodic hydrogen evolution reaction (HER) and anodic oxygen evolution reaction (OER) [1,2], which largely depend on highly efficient electrocatalysts. Pt-based metals and Ru/Ir-based compounds are considered to be the best HER electrocatalysts and OER electrocatalysts, respectively. However, the high cost and scarcity of these noble metal electrocatalysts severely limit their widespread applications [3]. Ternary pyrite-type cobalt phosphosulfide shows a high intrinsic HER activity in acidic solutions with a very low overpotential (*η*_10_) of 48 mV vs. RHE for *j* = −10 mA cm^−2^ and outstanding long-term operational stability [4]. However, due to the lack of stable electrocatalysts for acidic OER, electrolyzers that can operate in acidic media are obstacles to practical applications. Thus, high-performance HER catalysts in alkaline solutions are more suitable for industrial hydrogen production. During the past decades, high-efficiency and earth-abundant compounds, such as first-row (3D) transition metal sulfides [5,6,7], phosphides [8,9,10], and phosphosulfides, have shown their potential to serve as candidates for the HER as alternatives to precious noble metal-based electrocatalysts in alkaline solutions. Furthermore, various strategies to enhance the performance of HER have been explored, including surface and interface engineering [11], electronic and band structure engineering [12], strain engineering [13], conductivity engineering [14], and nanocomposites [15].

Previous works have demonstrated that for transition metal-based materials, higher phosphorus or sulfide contents will improve the HER catalytic activity [16,17]. Inspired by this idea, a new class of layered materials, metal trichal-cogenidophosphates (MTPs, with the chemical formula of MPX_3_ (M = Ni, Fe, Co, Mn, etc.; and X = S, Se)), have attracted attention due to their high nonmetal element ratio. Thus, they may serve as active sites during the HER [18,19,20,21,22]. However, the intrinsic limitation of MTPs for water splitting lies in the inert surfaces, and only the finite edge sites demonstrate catalytic activities [23,24,25]. To activate these inert surfaces, some strategies have been proposed, such as elemental doping [24,26] and high-entropy strategies [18]. However, the HER performance still requires further improvements. Another obstacle to the use of MTPs is their complex synthesis procedures, which usually require higher temperatures and long sintering times. The commonly used bulk MTPs are usually difficult to exfoliate, which largely hides the active edge sites and decreases the overall catalytic performance [27,28,29].

Herein, we proposed a simple two-step method to synthesize NiPS_3_ nanosheets (NSs) on a conductive substrate and investigated the HER performance of the as-prepared NSs and bulk powder materials. To further enhance the intrinsic catalytic activity, an edge amorphization strategy was performed by Ar etching, which gave the final Ar-NiPS_3_ an overpotential (*η*_10_) of 103.1 mV at a current density of 10 mA cm^−2^. The Ar-NiPS_3_ sample also exhibited a better OER activity (*η*_50_ = 278.9 mV vs. RHE and a Tafel slope of 66.5 mV dec^−1^). This may have been due to the easier oxidation of the peramorphic edge layers.

## 2. Results and Discussion

**Figure 1:**
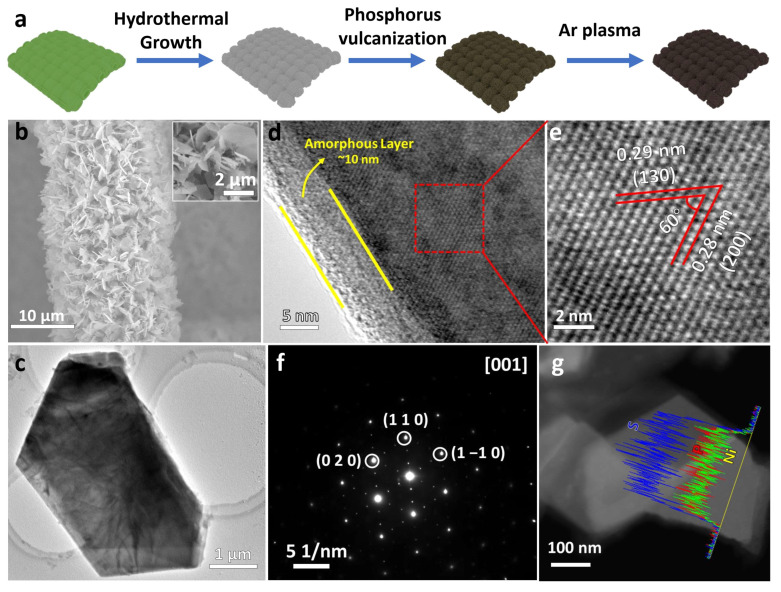
(**a**) Schematic illustration of the synthesis process for Ar-NiPS_3_; (**b**) SEM image and (**c**) TEM image of an Ar-etched NiPS_3_ nanosheet; (**d**) HRTEM image of an Ar-etched NiPS_3_ nanosheet with a ~10 nm amorphous layer; (**e**) enlarged HRTEM image and (**f**) SAED pattern of the basal plane; (**g**) EDS line scan profile of the nanosheet.

Self-supported monoclinic NiPS_3_ nanosheets were prepared on carbon cloth (CC) by a two-step process. Then, Ar plasma etching was conducted to modify the surface (Ar-NiPS_3_) (Figure 1a, details provided in the Experimental Section). To investigate the effect of synthesis conditions, different sintering temperatures were applied. As shown in Figures S1 and S2, phase-pure NiPS_3_ NSs were obtained between 450 °C and 550 °C. When the temperature was further increased to 650 °C, Ni_17_S_18_ became the main phase of the NSs and a higher temperature resulted in the collapse of the NSs (Figure S2d). To further determine the best sintering conditions, X-ray diffraction patterns and Raman spectra were obtained for different samples prepared under the temperature between 450 °C and 550 °C (Figures S3 and S4). No obvious differences were detected for any of the samples. NiPS_3_ powder was also prepared via a solid-state reaction for a comparison (Figure S5). Linear sweep voltammetry (LSV) curves for different samples prepared between 450 °C and 550 °C showed that the sample prepared at 470 °C showed the best HER performance (Figure S6). Thus, 470 °C was chosen as the optimal temperature to prepare NiPS_3_ NSs. To study the morphology and microstructure evolution of Ar-etched NiPS_3_ NSs, scanning electron microscopy (SEM) and transmission electron microscopy (TEM) were applied. The SEM and TEM images showed that Ar etching did not change the overall morphology of the NSs (Figure 1b,c), indicating their stability. High-resolution transmission electron microscopy (HRTEM) images showed representative interplanar spacings of 0.29 nm and 0.29 nm with an angle of 60°, which were, respectively, assigned to the (130) and (200) planes of monoclinic NiPS_3_ (Figure 1d,e). The corresponding selected area electron diffraction (SAED) pattern taken along the [0 0 1] zone axis is presented in Figure 1f, which was indexed to monoclinic NiPS_3_. This further revealed the single-crystal characteristics of the fabricated NiPS_3_ nanosheet arrays. A high-angle annular dark field–scanning transmission electron microscopy–energy-dispersive X-ray spectra (HAADF-STEM-EDS) line scan was used to investigate the elemental distribution and ratio of the nanosheet. The results revealed the homogeneous distribution of both Ni, P, and S elements across the whole nanoflower, and the ratio was about Ni:P:S = 1:1:3 (Figure 1g).

**Figure 2:**
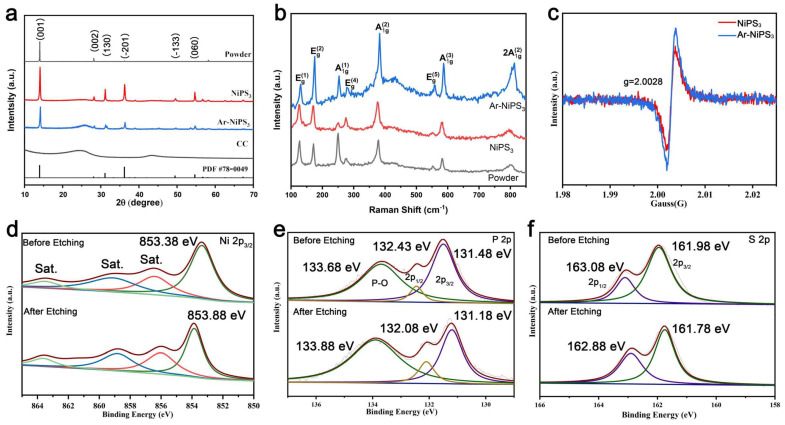
(**a**) XRD patterns of NiPS_3_ powder, NiPS_3_ NSs, Ar-NiPS_3_ NSs, and CC; (**b**) Raman spectra of NiPS_3_ powder, NiPS_3_ NSs, and Ar-NiPS_3_ NSs; (**c**) EPR spectra of NiPS_3_ NSs and Ar-NiPS_3_ NSs; XPS spectra of (**d**) Ni 2p, (**e**) P 2p, and (**f**) S 2p regions of NiPS_3_ NSs before and after Ar etching.

The crystal structures of the representative samples were characterized by X-ray diffraction (XRD) and Raman spectroscopy. As shown in Figure 2a, peaks at 13.9°, 28.1°, 31.1°, 36.2°, 49.5°, and 54.7° were observed for NiPS_3_ and Ar-NiPS_3_ (PDF #78−0499). To demonstrate the advantages of the proposed nanocrystallization strategy, NiPS_3_ powder was also prepared by a solid-state reaction, and it showed good crystallinity. The Raman spectra showed eight Raman-active phonon modes, in agreement with earlier work, which showed three out-of-plane A_1g_ modes and five in-plane E_g_ modes [30] (Figure 2b). According to the previous report, the A_1g_ ratio can be used to indicate the thickness of NiPS_3_ [31,32]. It showed clearly that Ar-NiPS_3_ had a much stronger A_1g_ phonon mode, indicating that Ar etching reduced the thickness of the NSs, thus exposing more active sites at the edges. We further conducted electron spin resonance (ESR) spectroscopy to study the influence of Ar etching. The ESR spectra showed that after Ar etching, more vacancies appeared in NiPS_3_ NSs, which may have produced more active sites in NSs, which further enhanced the catalytic activity [33,34,35] (Figure 2c). High-resolution X-ray photoelectron spectroscopy (XPS) was applied to gain insights into the surface chemical state of NiPS_3_ and Ar-NiPS_3_. For pristine NiPS_3_, the Ni 2p_3/2_ spectrum showed a main peak at 853.38 eV, which belonged to Ni^2+^ species. It also contained three other satellite peaks at 856.43, 859.13, and 863.53 eV (Figure 2d) [27]. After Ar etching, the Ni 2p_3/2_ peak underwent a shift to a higher binding energy (about 0.5 eV), indicating a higher valence state of Ni atoms in the NSs. In the P 2p and S 2p spectra, the peaks all shifted to lower binding energies after Ar etching (Figure 2e,f). (The peak at 133 eV in the P 2p spectrum belongs to the P-O bond, which was due to inevitable surface oxidation during synthesis [36,37]). Since XPS is a surface-sensitive detection technique, these results indicate the charge redistribution at the amorphous layer after Ar etching. According to the XPS spectra, electrons transferred from Ni to P and S, which produced high-valence Ni species. It has been shown that high-valence Ni undergoes an upshift in the d-band center of the catalytic system. A shallower *d*-band center will produce stronger binding between adsorbates and the surface of catalysts [38]. For HER in alkaline media, the rate-determining step is the Volmer step, which provides protons for other catalytic steps [1]. Electron-rich P and S sites provided sufficient electrons for reducing protons, which enhanced the HER performance of Ar-NiPS_3_ NSs [26]. From the analysis above, it could be speculated that Ar-etched NiPS_3_ nanosheets would demonstrate better catalytic performance by increasing the number of active sites at the edges of NSs. They also decreased the energy barrier of the reaction by modulating the electronic structure around active sites.

**Figure 3:**
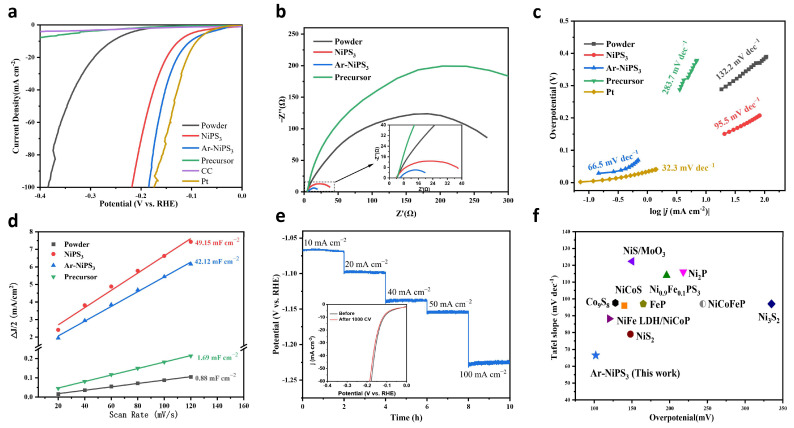
(**a**) iR-corrected LSV curves of different samples measured in 1 M KOH and (**b**) Nyquist plots of different samples; (**c**) corresponding Tafel slopes; (**d**) electrochemical double-layer capacitance of the corresponding samples of the as-prepared Ar-NiPS_3_, NiPS_3_ NS, NiPS_3_ powder, and Ni precursor; (**e**) stability test of Ar-NiPS_3_ NSs (LSV curves before and after 1000 CV are shown in the inset); (**f**) comparison of HER activities of Ar-NiPS_3_ and other representative electrocatalysts reported in refs. [19,43,44,45,46,47,48,49,50,51].

The HER performances of Ar-etched NiPS_3_ NSs and other samples were evaluated in 1 M KOH. We first investigated the influence of etching time. Upon increasing the etching time, the HER performance first increased and then decreased, and the sample etched with Ar plasma for 10 s demonstrated the best HER performance. Thus, we can conclude that the etching process should be kept at a moderate degree since upon increasing the etching time, the thickness of the amorphous layer increased, which may have provided more active sites. However, a thicker amorphous layer may also decrease the conductivity of the nanosheets and reduce the overall HER performance [39]. To further demonstrate the advantages of our nanocrystallization and etching strategy, the HER performances of different samples, including bulk NiPS_3_, NiPS_3_ NSs, and Ar-NiPS_3_, were further tested. As shown in Figure 3a, Pt wire showed the best HER performance and delivered a current density of 10 mA cm^−2^ at an overpotential (*η*_10_) of 89.2 mV. In contrast, NiPS_3_ powder showed the worst catalytic performance, with *η*_10_ = 256.6 mV. As expected, our self-standing NiPS_3_ NSs electrode demonstrated *η*_10_ = 125.2 mV, which was 51.2% lower than that of the bulk materials. Ar-NiPS_3_ showed enhanced HER performance with *η*_10_ = 103.1 mV. According to the XPS and HRTEM analysis, the amorphous layer was responsible for this enhancement due to charge redistribution around the active sites. The amorphous layer also provided abundant active sites at edges [40]. A comparison of the HER activities of different samples further proved the effectiveness of the proposed nanocrystallization and etching strategy. Furthermore, the electrochemical impedance spectroscopy (EIS) results indicated a smaller charge transfer resistance (*R*_ct_) of Ar-NiPS_3_ than other samples, suggesting faster HER kinetics (Figure 3b and Table S1) [41]. The Tafel slope is a commonly used indicator for the rate-determining step. As shown in Figure 3c, Ar-NiPS_3_ demonstrated a Tafel slope of 66.5 mV dec^−1^, which was much lower than that of NiPS_3_ (95.5 mV dec^−1^) and bulk NiPS_3_ powder (132.2 mV dec^−1^). This indicated that the HER occurred on the Ar-NiPS_3_ via the Volmer–Heyrovsky mechanism and that the Heyrovsky reaction was the rate-determining step [1]. Moreover, the electrochemically active surface area (ECSA) of different samples was estimated by comparing the double-layer capacitance (C_dl_) with the cyclic voltammetry (CV) measurements (Figure S7). The results showed that nanocrystallization significantly increased the ECSA of NiPS_3_, which was about 47 times higher than that of the powder materials. Ar etching further enhanced the ECSA from 42.12 mF cm^−2^ to 49.15 mF cm^−2^. This observation indicated that the Ar-NiPS_3_ electrode exposed more catalytic active sites than other electrodes, which significantly increased its HER performance [42] (Figure 3d). Stability is another important indicator for obtaining promising electrocatalysts. As shown in Figure 3e, the HER performance of Ar-NiPS_3_ did not show obvious degradation after long-term stability tests under different current densities and 1000 CV measurements, demonstrating its excellent stability (inset in Figure 3e). The excellent HER performance also made Ar-NiPS_3_ a superior catalyst to previously reported noble-metal-free HER catalysts in alkaline media (Figure 3f and Table S2).

**Figure 4:**
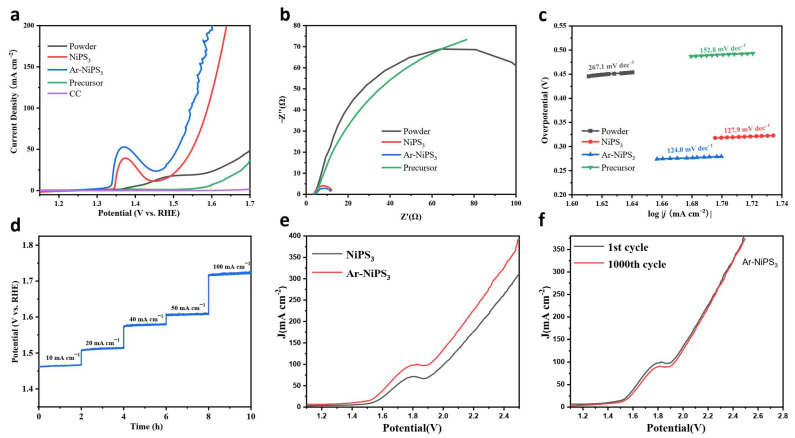
Electrochemical OER performance of different samples: (**a**) iR-corrected LSV curves of different samples measured in 1 M KOH; (**b**) Nyquist plots of different samples; (**c**) corresponding Tafel slopes; (**d**) stability test of Ar-NiPS_3_ NSs under different current densities; (**e**) performance of the full water-splitting device using the Ar-NiPS_3_ and NiPS_3_ electrode as both the anode and cathode; (**f**) stability test before and after 1000 CV cycles of Ar-NiPS_3_.

Considering the excellent HER performance of Ar-NiPS_3_ NSs, we also measured the OER activities of the modified samples and investigated the overall water-splitting performance. As shown in Figure 4a, the Ar-etched NiPS_3_ electrode demonstrated the best OER activity, with an overpotential of 278.9 mV, reaching a current density of 50 mA cm^−2^. NiPS_3_, NiPS_3_ powder, and Ni precursor required higher overpotentials of 317.86 mV, 475.1 mV, and 490.0 mV, respectively, to deliver the same current density. The EIS results showed that NiPS_3_ and Ar-NiPS_3_ had lower *R*_ct_ values than the other samples, which may have been caused by the fully-exposed active sites on the nanosheets (Figure 4b and Table S3). The Tafel slope obtained by extrapolating the linear region of overpotential vs. log *j* (Figure 4c) was 124.0 mV dec^−1^ for Ar-NiPS_3_ (after iR correction) and 127.9, 158.2, and 267.1 mV dec^−1^ for the NiPS_3_, Ni precursor, and NiPS_3_ bulk materials, respectively. This indicated that Ar-etched NiPS_3_ demonstrated faster OER kinetics. Ar-NiPS_3_ still exhibited satisfying stability during long-term tests. We studied the chemical state of Ar-NiPS_3_ after OER tests. For transition metal phosphides, sulfides, and nitrides, the final active species are the corresponding in-situ-derived (hydro)oxides [52]. After the OER test, the surface of Ar-NiPS_3_ mainly turned into Ni-O species (Figure S8a), and the peaks of P 2p almost disappeared (Figure S8b), while that of S-O obviously increased (Figure S8c). Metal–oxygen (M-O) bonds became the main peak in the O 1s spectrum, which indicated the surface of the sample was totally oxidized under an anodic current. The surface metal oxides could also accelerate the adsorption of OH^−^, which further enhanced the OER (Figure S8d) [53]. We also investigated the full water-splitting performance with Ar-NiPS_3_ serving as both the cathode and anode in a two-electrode system. As shown in Figure 4e, the Ar-NiPS_3_//Ar-NiPS_3_ electrode couple demonstrated enhanced full water-splitting activity compared to the NiPS_3_//NiPS_3_ electrode couple and excellent stability without obvious degradation after the stability test.

## 3. Materials and Methods

Materials. Nickel(II) nitrate hexahydrate (Ni(NO_3_)_2_·6H_2_O), ammonium fluoride (NH_4_F), high-purity sulfur powder, and red phosphorus powder were obtained from Alfa Aesar. Urea was purchased from Sinopharm Chemical Regent Co., Ltd. Carbon cloth (CC) was provided by Shanghai Hesen Corp. Ultrapure water (>18.25 MΩ cm) was obtained from an Evoqua system. Commercial Pt film catalysts were purchased from Aladdin. All chemicals were of analytical grade and used as-received without further treatment.

Synthesis of Ni(OH)F NS/CC precursors. The Ni(OH)F precursors were prepared by a simple hydrothermal method. Briefly, Ni(NO_3_)_2_·6H_2_O was weighed in stoichiometric amounts (2 mmol). Then, NH_4_F (5 mmol), urea (10 mmol), and Ni(NO_3_)_2_·6H_2_O were dissolved in deionized water (40 mL) with continuous stirring for 30 min. The as-obtained homogeneous solution was then transferred to a 50 mL Teflon-lined stainless steel autoclave. CC was cut into small pieces (with dimensions of 2 cm × 3 cm) and cleaned by sonication sequentially in acetone, water, and ethanol for 20 min each to ensure a well-cleaned surface for use. A piece of cleaned CC was placed in an autoclave at 120 °C for 6 h. When the autoclave cooled naturally to room temperature, the samples were taken out and washed with deionized water several times before vacuum drying overnight at 70 °C.

Synthesis of the NiPS_3_ NS/CC electrode. A piece each of Ni(OH)F NS/CC (2 cm × 3 cm), sulfur powder (3782 mg), and red phosphorus powder (1218 mg) were placed in a corundum porcelain boat. Sulfur powder and phosphorous powder were placed upstream. Then, the porcelain boat was pushed into the heating zone in the middle of the tube furnace. High-purity argon gas was poured in to flush the furnace tube for 30 min to exhaust the air in the tube (300 sccm). After that, high-purity argon gas was continuously injected (100 sccm). The next step was to increase the temperature to 470 °C, at a heating rate of 5 °C/min. After holding at 470 °C for 2 h, it was cooled down naturally. After cooling, the black sample was removed, which was the NiPS_3_ nanosheet catalytic electrode. The loading of catalyst on the CC substrate was about 6.8 mg/cm^2^.

Preparation of Ar plasma-treated NiPS_3_ nanosheets. A piece of NiPS_3_ NS/CC (2 cm × 3 cm) was fixed on a silicon chip and treated by Ar plasma. The power of argon plasma and flow rate of Ar were fixed at 150 W and 100 sccm, respectively, with different irradiation times (0 s, 5 s, 10 s, 15 s). The NiPS_3_ NS/CC (Ar-NiPS_3_) plasma treated for 10 s was used for detailed studies.

Material Characterization: The crystal structure of the samples was determined by an X’Pert Pro Super diffractometer with Cu-Kα radiation (λ = 1.54178 Å). Field-emission scanning electron microscope (SEM) images were obtained on a Hitachi SU8010 SEM. TEM, HRTEM, AC-HAADF-STEM, and elemental maps were conducted on a JEM-ARM200CF microscope operating at 200 kV. XPS spectra were collected on an ESCALAB MK II with an Al Kα excitation source. Raman spectra were measured on a Renishaw InVia Reflex Raman microscope. The electron paramagnetic resonance (EPR) spectra were obtained on a Bruker A300-10/12.

Electrochemical Measurements: All electrochemical measurements in this work were carried out on a CHI 760E electrochemical workstation in 1 M KOH. HER performances for each sample were measured with a three-electrode system at room temperature. The prepared samples were used as the working electrodes directly. A graphite rod (Alfa Aesar, Shamghai, China, 99.9995%) and an *Hg*/HgO electrode were used as the counter electrode and the reference electrode, respectively. LSV curves were obtained at a scan rate of 2 mV s^−1^. In this work, all measured potentials were referred to the reversible hydrogen electrode (RHE) using the following Equation (1)

(1) $$E_{\mathrm{RHE}}=E_{\mathrm{Hg}/\mathrm{HgO}}+0.098V+0.059\times pHE_{\mathrm{RHE}}$$

EIS measurements were carried out within a frequency range from g 0.01 Hz to 10^6^ Hz for all electrolytes. The series resistance (*R_s_*) obtained from EIS measurements were used to correct the polarization measurement with Equation (2)

(2) $$E_{corrected}=E_{uncorrected}-iR_{s}$$

CV test was used to perform the stability test with the scan range of −0.6 to 0 V versus RHE, and the scan rate was set as 50 mV s^−1^. The ECSA was estimated from the corresponding electrochemical double-layer capacitances (Cdl) with CV measurements. CV curves were collected in a non-Faradaic region at various scan speeds ranging from 20 to 120 mV s^−1^ at the potential range of −0.62–−0.77 V vs. RHE.

## 4. Conclusions

Here, a two-step method was proposed to develop high-efficiency water-splitting electrodes by modifying the edge sites of NiPS_3_ nanosheets. The HER, OER, and overall water-splitting performances were studied. The self-standing NiPS_3_ electrode provided abundant active sites after Ar etching by forming a uniform amorphous layer at the edges of NiPS_3_ nanosheets. This reduced the charge transfer resistance during the catalytic process and delivered *η*_10_ = 103.1 mV and *η*_50_ = 278.9 mV for the HER and OER, respectively. The Ar-NiPS_3_ electrode also demonstrated better catalytic activity and stability during overall water splitting. This study provides an efficient electrode for water splitting and also provides a promising strategy for the further modification of transition metal-based electrocatalysts.

## Data Availability

Samples of the compounds are available from the authors.

## Supplementary Materials

The following supporting information can be downloaded at: https://www.mdpi.com/article/10.3390/molecules28010315/s1. References [54,55] are cited in Supplementary Materials. Figure S1. The XRD patterns of the as-prepared NiPS3 NS/CC samples at different temperatures. Figure S2, The SEM image of the as-prepared NiPS3 NS/CC samples at 350 ℃ (a), 450 ℃ (b), 550 ℃ (c), 650 ℃ (d) (Scale bar: 50 μm), Figure S3. The XRD patterns of the as-prepared NiPS3 NS/CC samples at 450 ℃ to 550 ℃, Figure S4. Raman spectra of the NiPS3 NS/CC samples prepared under different temperatures and the NiPS3 Powder sample, Figure S5. The SEM image of the as-prepared NiPS3 powder sample (inset showed the enlarged image of the powder, which showed layered structure of NiPS3), Figure S6. Polarization curves of different sample prepared under different temperatures, Figure S7. CV curves of (a) Ar-NiPS3 NS/CC, (b) NiPS3 NS/CC, (c) NiPS3 Powder and (d) Ni Precursor under different scan rates, in the potential range from −0.62 V to −0.77 V vs. RHE, Figure S8. XPS spectra of (a) Ni 2p3/2, (b) P 2p, (c) S 2p, and (d) O 1s after OER test, Table S1. HER data obtained from the corresponding LSV curves, Table S2. OER data obtained from the corresponding LSV curves, Table S3. Comparison of some reported HER electrocatalysts in 1 M KOH.
