# Supplementary material for: Efficient Hydrogen and Oxygen Evolution Catalysis Using 3D-Structured Nickel Phosphosulfide Nanosheets in Alkaline Media
Source: Molecules. 2022 Dec 30;28(1):315. doi: 10.3390/molecules28010315 (PMC9822134; doi:10.3390/molecules28010315)
Supplement: Supplementary file 1 [file molecules-28-00315-s001.zip › molecules-2081260-supplementary.pdf]

Supporting information

Efficient Hydrogen and Oxygen Evolution Catalysis Using 3D-Structured Nickel Phosphosulfide Nanosheets in Alkaline Media

Lei Lin[1], Qiang Fu[1,*], Junbei Hu[1], Ran Wang[2], Xianjie Wang[1,*]

[1] School of Physics, Harbin Institute of Technology, Harbin 150001, China

[2] National Key Laboratory of Science and Technology on Advanced Composites in Special Environments, Harbin Institute of Technology, Harbin 150001, China

[*] Correspondence: fqianghit@163.com (Q.F.); wangxianjie@hit.edu.cn (X.-J.W.)

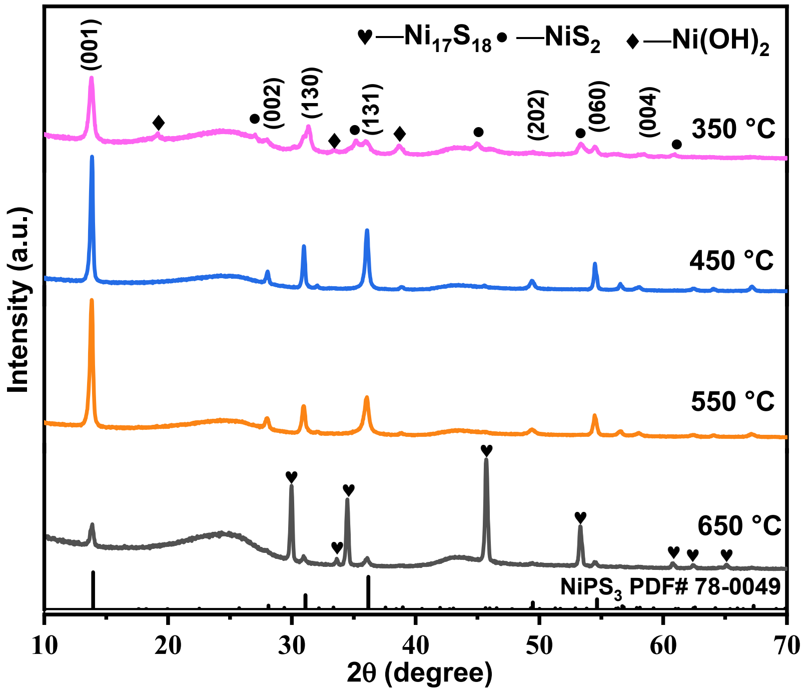

Figure S1. The XRD patterns of the as-prepared $NiPS_3$ NS/CC samples at different temperatures.

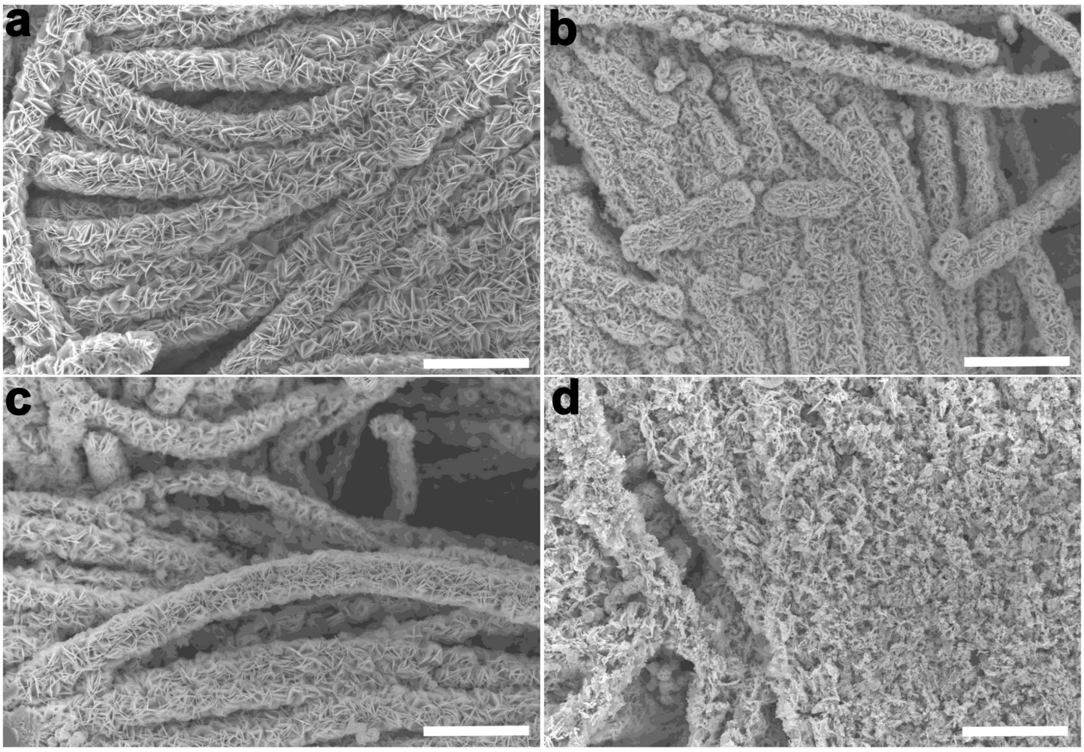

Figure S2. The SEM image of the as-prepared $NiPS_3$ NS/CC samples at 350℃(a), 450℃(b), 550℃(c), 650℃(d) (Scale bar: 50 μm).

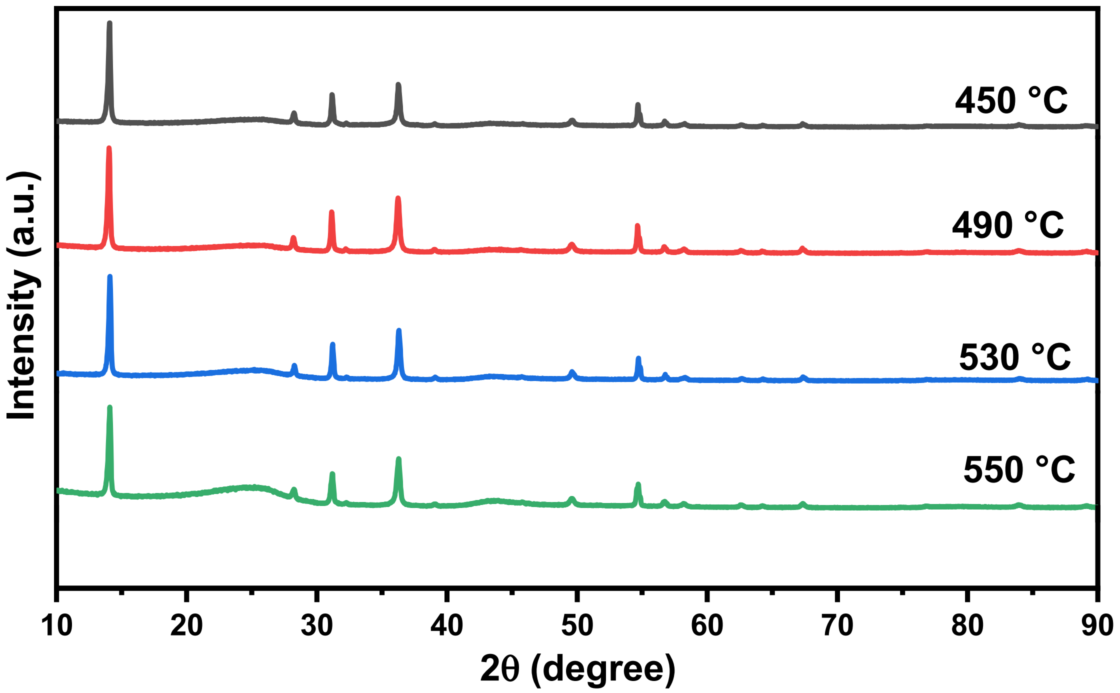

Figure S3. The XRD patterns of the as-prepared $NiPS_3$ NS/CC samples at 450 ℃ to 550 ℃.

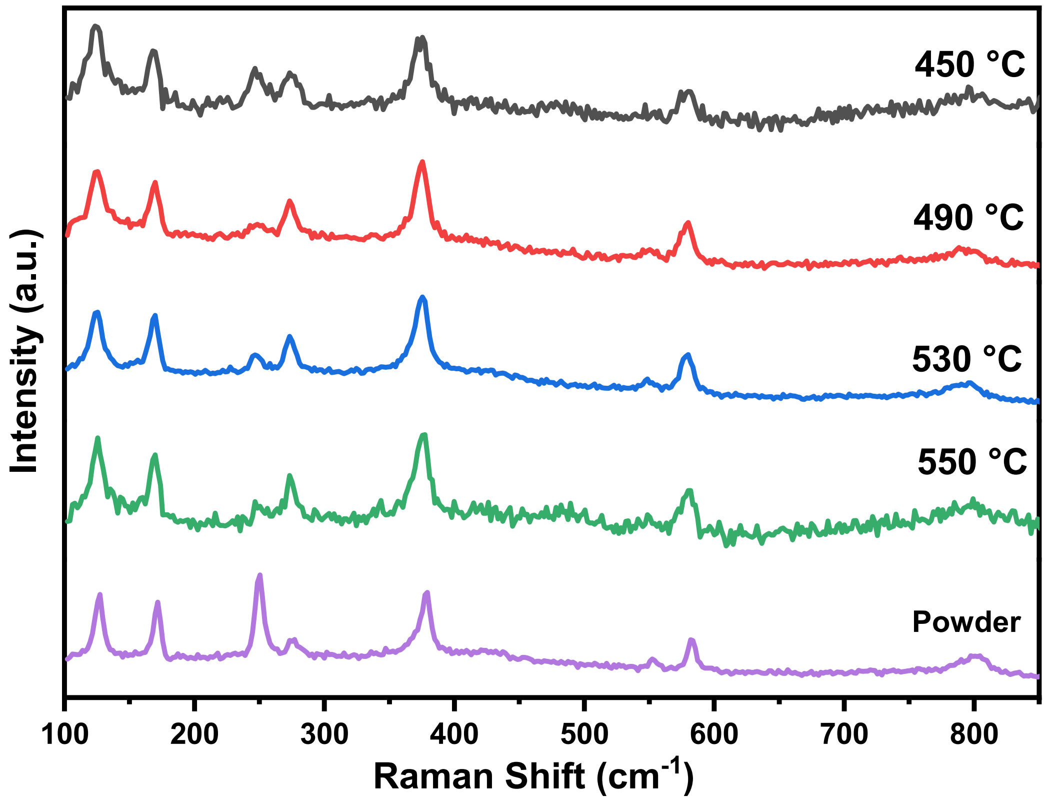

Figure S4. Raman spectra of the $NiPS_3$ NS/CC samples prepared under different temperatures and the $NiPS_3$ Powder sample

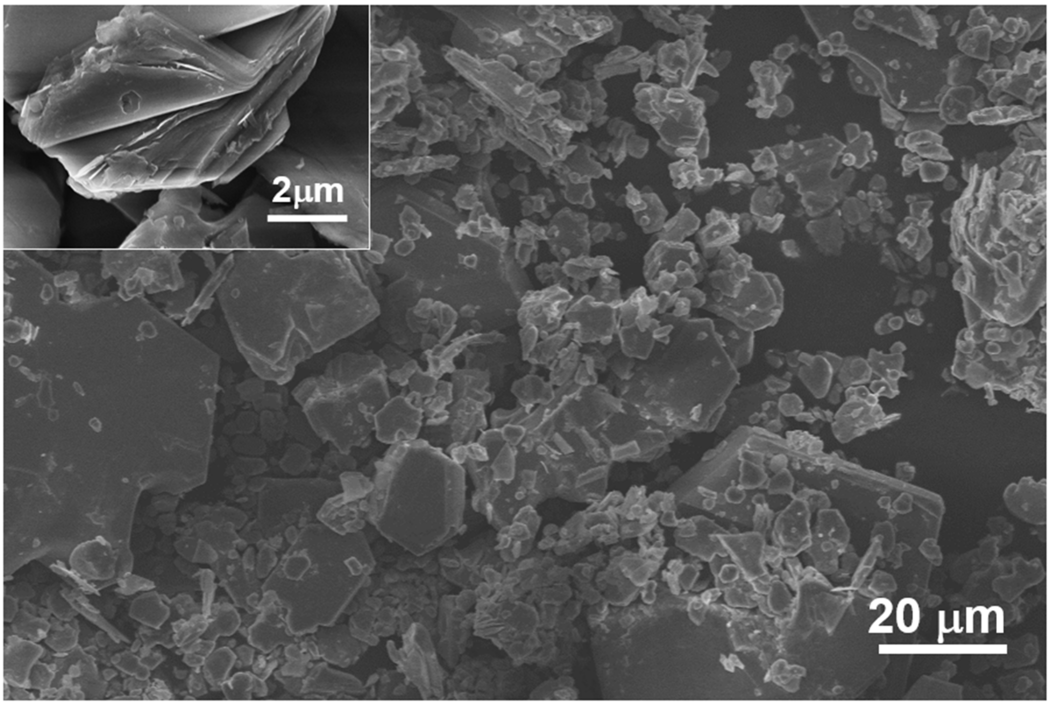

Figure S5. The SEM image of the as-prepared $NiPS_3$ powder sample (inset showed the enlarged image of the powder, which showed layered structure of $NiPS_3$).

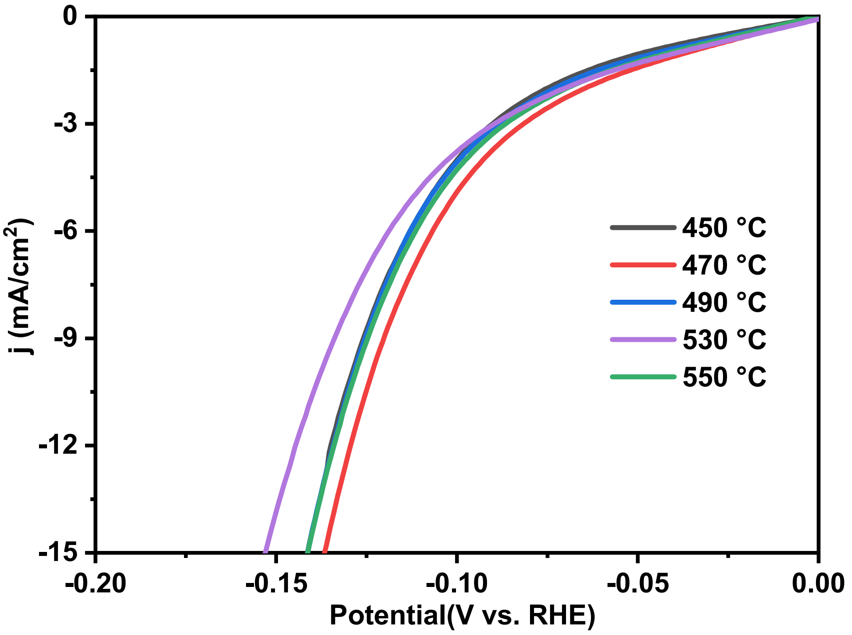

Figure S6. Polarization curves of different sample prepared under different temperatures.

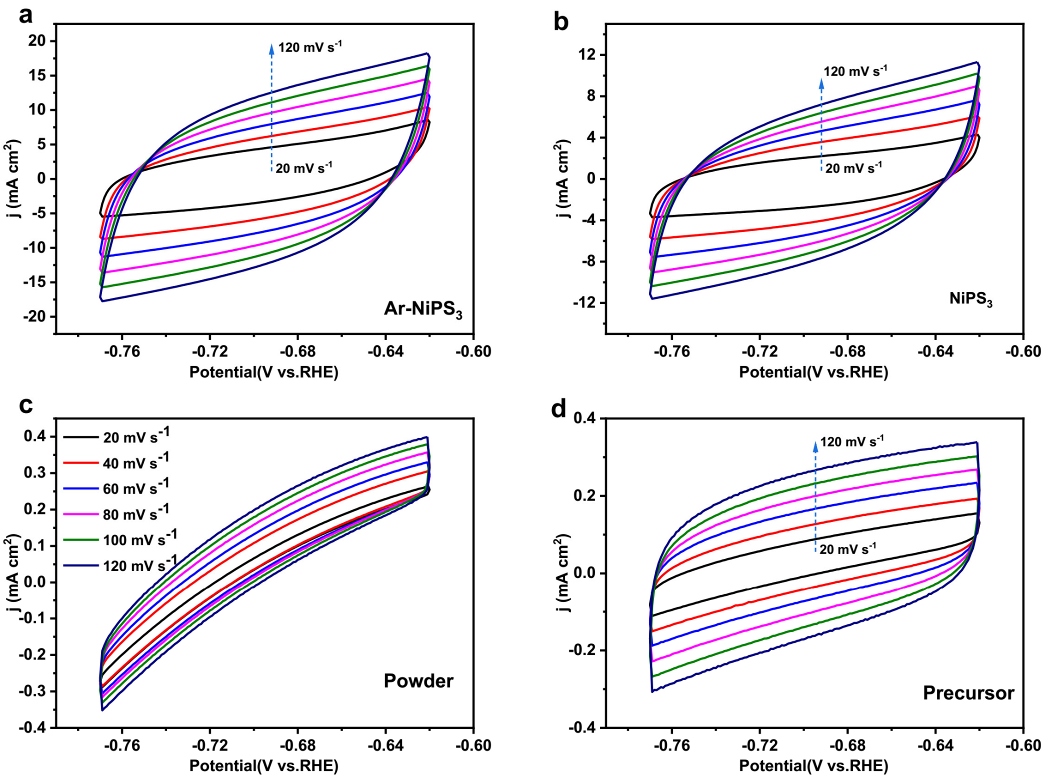

Figure S7. CV curves of (a) Ar-$NiPS_3$ NS/CC, (b) $NiPS_3$ NS/CC, (c) $NiPS_3$ Powder and (d)Ni Precursor under different scan rates, in the potential range from -0.62 V to -0.77 V vs. RHE. These data were used to generate the plots showing the extraction of the C_{dl} for different samples shown in Figure 3d.

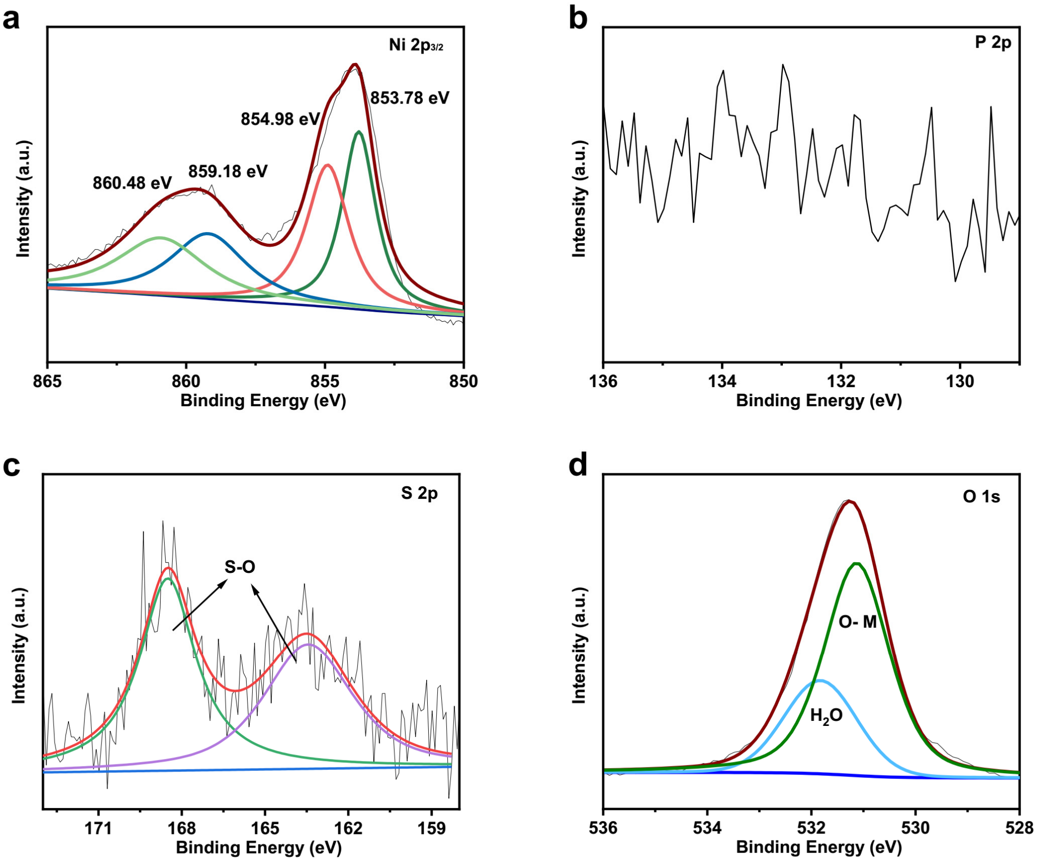

Figure S8. XPS spectra of (a) Ni $2p_{3/2}$,(b) P 2p, (c) S 2p, and (d) O 1s after OER test.

Table S1. HER data obtained from the corresponding LSV curves.

**Catalyst**	**Tafel slope (mV dec^{-1})**	**Overpotential (mV) at 10 mA cm^{-2}**	**Electrolyte**
Ni-precursor	283.7	436.41	1 M KOH
$NiPS_3$ Powder	132.2	256.57	1 M KOH
$NiPS_3$ NS	95.5	125.18	1 M KOH
Ar-$NiPS_3$ NS	66.5	103.10	1 M KOH

Table S2. OER data obtained from the corresponding LSV curves.

**Catalyst**	**Tafel slope (mV dec^{-1})**	**Overpotential (mV) at 50 mA cm^{-2}**	**Electrolyte**
Ni-precursor	152.8	490.01	1 M KOH
$NiPS_3$ Powder	267.1	475.08	1 M KOH
$NiPS_3$ NS	127.9	317.86	1 M KOH
Ar-$NiPS_3$ NS	124.0	278.89	1 M KOH

Table S3. Comparison of some reported HER electrocatalysts in 1 M KOH.

**Catalyst**	**Tafel slope ($mV\ dec^{-1}$)**	**Overpotential (mV) at 10 $mA\ cm^{-2}$**	**Ref.**
Ni-Co-S/CF	96	140	[1]
NiS_2	79	148	[2]
Ni_2P	116	218	[3]
$Ni_{0.9}Fe_{0.1}PS_3$/Mxene	114	196	[4]
Ni_3S_2	97	335	[5]
NiS/MoO_3/NF	122.3	150	[6]
NiFe-LDH/NiCoP/NF	88.2	120	[7]
Co_9S_8	97.6	128	[8]
FeP	97	165	[9]
MoS_2/NiS	97	244	[10]
**Ar-$NiPS_3$**	**66.5**	**103.10**	**This Work**

References:

1. Yao, M.; Hu, H.; Sun, B.; Wang, N.; Hu, W.; Komarneni, S., Self-Supportive Mesoporous Ni/Co/Fe Phosphosulfide Nanorods Derived from Novel Hydrothermal Electrodeposition as a Highly Efficient Electrocatalyst for Overall Water Splitting. *Small* **2019,** 15, (50), 1905201.
2. Luo, P.; Zhang, H.; Liu, L.; Zhang, Y.; Deng, J.; Xu, C.; Hu, N.; Wang, Y., Targeted synthesis of unique nickel sulfide (NiS, NiS_2) microarchitectures and the applications for the enhanced water splitting system. *ACS Appl. Mater. Interfaces* **2017,** 9, (3), 2500-2508.
3. Liang, Q.; Zhong, L.; Du, C.; Luo, Y.; Zhao, J.; Zheng, Y.; Xu, J.; Ma, J.; Liu, C.; Li, S., Interfacing epitaxial dinickel phosphide to 2D nickel thiophosphate nanosheets for boosting electrocatalytic water splitting. *ACS nano* **2019,** 13, (7), 7975-7984.
4. Du, C.-F.; Dinh, K. N.; Liang, Q.; Zheng, Y.; Luo, Y.; Zhang, J.; Yan, Q., Self-Assemble and In Situ Formation of $Ni_{1-x}Fe_xPS_3$ Nanomosaic-Decorated MXene Hybrids for Overall Water Splitting. *Advanced Energy Materials* **2018,** 8, (26), 1801127.
5. Jiang, N.; Tang, Q.; Sheng, M.; You, B.; Jiang, D.-e.; Sun, Y., Nickel sulfides for electrocatalytic hydrogen evolution under alkaline conditions: a case study of crystalline NiS, NiS_2, and Ni_3S_2 nanoparticles. *Catalysis Science & Technology* **2016,** 6, (4), 1077-1084.
6. Jin, L.; Xu, H.; Wang, C.; Wang, Y.; Shang, H.; Du, Y., Multi-dimensional collaboration promotes the catalytic performance of 1D MoO_3 nanorods decorated with 2D NiS nanosheets for efficient water splitting. *Nanoscale* **2020,** 12, (42), 21850-21856.
7. Zhang, H.; Li, X.; Hähnel, A.; Naumann, V.; Lin, C.; Azimi, S.; Schweizer, S. L.; Maijenburg, A. W.; Wehrspohn, R. B., Bifunctional Heterostructure Assembly of NiFe LDH Nanosheets on NiCoP Nanowires for Highly Efficient and Stable Overall Water Splitting. *Adv. Funct. Mater.* **2018,** 28, (14), 1706847.
8. Du, F.; Shi, L.; Zhang, Y.; Li, T.; Wang, J.; Wen, G.; Alsaedi, A.; Hayat, T.; Zhou, Y.;

Zou, Z., Foam–like Co_9S_8/Ni_3S_2 heterostructure nanowire arrays for efficient bifunctional overall water–splitting. *Appl. Catal.B: Environ* **2019,** 253, 246-252.

9. Yao, S.; Forstner, V.; Menezes, P. W.; Panda, C.; Mebs, S.; Zolnhofer, E. M.; Miehlich, M. E.; Szilvási, T.; Kumar, N. A.; Haumann, M., From an Fe_2P_3 complex to FeP nanoparticles as efficient electrocatalysts for water-splitting. *Chem.Sci.* **2018,** 9, (45), 8590-8597.

10. Qin, Q.; Chen, L.; Wei, T.; Liu, X., MoS_2/NiS Yolk–Shell Microsphere-Based Electrodes for Overall Water Splitting and Asymmetric Supercapacitor. *Small* **2019,** 15, (29), 1803639.
